# Inhalable dry powder product (DPP) of mRNA lipid nanoparticles (LNPs) for pulmonary delivery

**DOI:** 10.1007/s13346-023-01402-y

**Published:** 2023-08-01

**Authors:** Ashish Sarode, Priyal Patel, Natalia Vargas-Montoya, Ayed Allawzi, Alisa Zhilin-Roth, Saswata Karmakar, Lianne Boeglin, Hongfeng Deng, Shrirang Karve, Frank DeRosa

**Affiliations:** 1 mRNA Center of Excellence, 200 West Street, MA 02451 Sanofi Waltham, USA; 2 Translate Bio, Lexington, MA USA

**Keywords:** mRNA, Lipid nanoparticle (LNP), Dry powder, Spray drying, Pulmonary, Mannitol, Leucine

## Abstract

Pulmonary delivery of mRNA via inhalation is a very attractive approach for RNA-based therapy for treatment of lung diseases. In this work, we have demonstrated successful development of an mRNA-lipid nanoparticle (LNP) dry powder product (DPP), wherein the LNPs were spray dried using hydroalcoholic solvent along with mannitol and leucine as excipients. The desired critical attributes for the DPP were accomplished by varying the excipients, lipid composition, concentration of LNPs, and weight percentage of mRNA. Leucine alone or in combination with mannitol improved the formulation by increasing the mRNA yield as well as decreasing the particle size. Intratracheal administration of the DPP in mice resulted in luciferase expression in the trachea and lungs indicating successful delivery of functional mRNA. Our results show formulation optimization of mRNA LNPs administered in the form of DPP results in an efficacious functional delivery with great promise for future development of mRNA therapeutics for lung diseases.

**Graphical Abstract:**

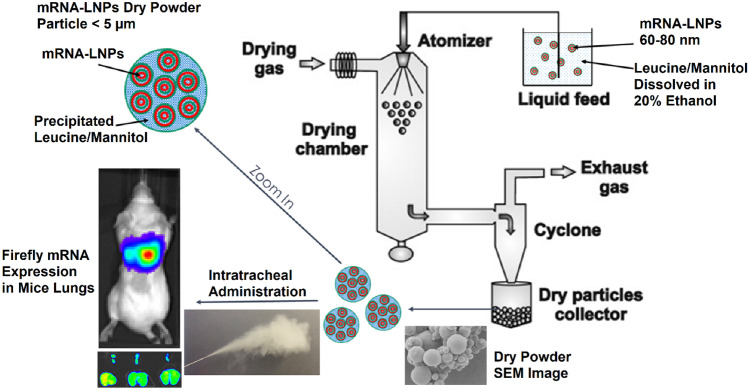

**Supplementary Information:**

The online version contains supplementary material available at 10.1007/s13346-023-01402-y.

## Introduction

The rapid approval and remarkable success of Comirnaty (BNT162b2) and Spikevax (mRNA-1273) have demonstrated the clinical validation of mRNA as a new class of highly efficacious nucleic acids in the field of vaccines [1]. The mechanism by which an mRNA vaccine acts involves delivery of synthetically transcribed mRNA molecules that encodes for a viral protein to the human host cells [2]. The host cells then produce the viral protein that leads to adaptive immune response to fight the corresponding viral infection [3]. The application of mRNA extends beyond vaccines in the areas of protein replacement therapy for genetic diseases, gene editing, and oncology applications, as well as others [4]. Therapeutic treatments for liver and lung diseases are being developed by delivering synthetic mRNA to encode for and replace a missing or non-functional protein to the corresponding target cells via intravenous and inhalable routes, respectively [5–7]. Delivering mRNA to either the liver or lung for therapeutic application has its own benefits and challenges [8]. Since the focus of this work is on developing dry powder (DP) formulations for inhalation, mRNA therapeutics has been discussed in the context of pulmonary delivery.

A pulmonary route of administration for mRNA may be preferred due to numerous advantages. Delivering inhalable medication to the lungs is a non-invasive method and offers direct access to upper and lower airway epithelial cells as well as the alveoli and lung parenchyma along with hundreds of square meters of surface area rich in vasculature for fast distribution [9]. Successful delivery of mRNA to the target cells in the lungs may result in the successful treatment of pulmonary diseases such as acute respiratory distress syndrome (ARDS), cystic fibrosis (CF), lung cancer, pulmonary alveolar proteinosis (PAP), pulmonary arterial hypertension (PAH), and primary ciliary dyskinesia (PCD) [10].

Despite of its inherent advantages, successful lung delivery requires lot of work around optimization of both the formulation and inhalation devices [11]. Nebulizers, pressurized metered dose inhalers (pMDIs), and dry powder inhalers (DPIs) are three main types of inhalation devices that are used for drug administration to the lungs [12]. Although nebulizers can generate fine mist that exhibits efficient deposition deep into the lung tissue, such devices are less patient friendly primarily due to the longer duration of inhalation and difficulty in operating [13]. In addition, achieving and maintaining a high rate of nebulization as well as maintaining critical quality attributes and stability of the mRNA-LNP product throughout the inhalation period can be challenging [14]. On the other hand, pMDIs are easy to handle and are more reliable due to their consistent metering leading to accurate dosing [15]. However, they also come with compliance issues due to a requirement of hand-lung coordination by the patient [16]. In addition, there are efforts to reduce the carbon footprint of pMDIs as they require greenhouse gases like hydrofluoroalkanes as propellants to release the required dose at higher speed [17]. DPIs are the most advanced inhalation devices for lung delivery and can overcome some of the challenges associated with nebulizers and pMDIs [18]. They are less expensive, portable, do not require any propellants for delivery, can deliver higher doses in shorter period, can be actuated by breathing, and are easy to operate [19, 20]. There are several DPI devices available in the market such as Rotahaler, Diskus, Spiros, Turbuhaler, and Clickhaler that discharge DPP to breathe in easily by the patient [21]. Furthermore, DPP affords higher stability due to its solid state, which can be advantageous for mRNA delivery [22, 23].

The performance of DPP mostly depends on the number of particles deposited in the target areas of the lungs; undeposited particles are either exhaled out or swallowed showing no efficacy or unwanted side effects. Although there are several factors that can influence movement of DPP particles into the airways, aerodynamic particle size primarily determines the site and extent of deposition [24]. Ideally, the mass median aerodynamic diameter (MMAD) of DPP particles should be below 5 µm as particles in the range of 1 to 3 µm exhibit the highest deposition in the central and peripheral airways leading to sedimentation and subsequent absorption, whereas particles below 1 µm and above 5 µm are exhaled out or swallowed, respectively [25]. Other characteristics that can also play some role in DPP flow in the airways include bulk and tap density, moisture content, rate of water absorption, flowability, and surface area of the particles [26].

Spray drying, which involves atomization of solution into droplets followed by rapid evaporation, is a commonly used, economical and an established technique to manufacture the DPP for various modalities such as small molecules, peptides, and proteins. The operation is continuous, scalable, suitable for heat-sensitive material and can produce robust, consistent DPP with desired formulation characteristics [27]. Various sugars such as lactose and mannitol are commonly used as carrier excipients to facilitate the spray drying process [28]. Mannitol is an attractive excipient for spray drying of mRNA formulations showing beneficial properties which include (i) altering the effect on the viscoelastic properties associated to phlegm, (ii) increasing water content driven by osmotic gradient, (iii) less hygroscopic compared to some other sugars like lactose, and (iv) stability as a non-reducing sugar with absence of any aldehyde group. Recently, amino acids such as leucine, valine, isoleucine, and trileucine have been utilized to improve aerosolization properties of inhalable microparticles by increasing the dispersibility, decreasing agglomeration, and decreasing the MMAD of DPP [26, 29]. Inhalable formulations of mRNA with peptides or exosomes have been demonstrated previously [8, 30]. To the best of our knowledge, reports of excipient screening and optimization of formulation properties and aerosol performance of spray dried mRNA LNPs are rare. In continuation of our efforts of developing dry powder formulation for mRNA delivery [31], herein, we report successful formulation optimization work of spray drying two types of LNP formulations in hydroalcoholic solutions made using novel ionizable lipids in combinations with mannitol and leucine as excipients. We have designed novel ionizable lipid families based of phenolic acid and citric acid cores that have shown promise in pulmonary delivery of mRNA [32, 33]. In this work, we have evaluated two such novel ionizable lipids: lipid A based on phenolic acid core and lipid B based on citric acid core (Fig. S1). Both lipids have ester linkers, making them potentially biodegradable and thus a lower risk of safety concerns. We demonstrate functional dry powder formulation of mRNA-LNPs with desired characteristics can be successfully formulated using spray drying technique by optimizing the solvents, excipient combinations, processing parameters, and LNP composition.

## Experimental section

### Materials

mRNAs were synthesized by in vitro transcription employing RNA polymerase with a plasmid DNA template encoding the gene using unmodified nucleotides, which was followed by the addition of a 5’ cap structure (Cap 1) and a 3’ poly(A) tail. 1,2-dimyristoyl-rac-glycero-3-methoxypolyethylene glycol-2000(DMG-PEG2000), 1,2-dioleoyl-sn-glycero-3-phosphoethanolamine (DOPE), and 1,2-dierucoyl-sn-glycero-3-phosphoethanolamine (DEPE) were purchased from NOF America Corporation (White Plains, NY, USA). Cholesterol, 1,2-dipalmitoyl-sn-glycero-3-phosphocholine (DPPC), leucine, trileucine, proleucine, and glycine were obtained from Millipore Sigma (Burlington, MA, USA). Mannitol, ethanol, and acetonitrile were purchased from Fisher Scientific. Citrate buffer pH 4.5 and sodium chloride solution were purchased from Boston Scientific (Marlborough, MA, USA).

### Preparation of mRNA-LNP-based DPP

Preparation of mRNA/lipid nanoparticle (LNP) formulations was performed as described earlier [34]. Briefly, an ethanolic solution of a mixture of lipids (ionizable lipid, phosphatidylethanolamine, cholesterol, and polyethylene glycol-lipid) was combined with an aqueous buffered solution of target mRNA at an acidic pH under controlled conditions to yield a suspension of uniform LNPs. The resultant mixture of ethanol and aqueous phase was exchanged into the suitable solution using a 300 kDa membrane (Millipore Pellicon) on a TFF system. Briefly, the resultant mix of lipids and mRNA was first concentrated via ultrafiltration followed by 6X diafiltration volumes with the final formulation solution. For spray drying, solutions of different excipients were added to the LNP suspensions resulting in final solutions at 20% ethanol concentration and spray dried on a Buchi-290 using parameters as shown in Table 1. The initial excipient screening evaluations were performed on LNP formulations with molar compositions of DMG-PEG-2000: ionizable lipid A: cholesterol: DOPE (5:40:25:30) and DMG-PEG-2000: ionizable lipid B: cholesterol: DOPE (3:40:25:32) with the ionizable lipid to mRNA weight ratio of 10 and 8 for lipid A and lipid B, respectively. Additional evaluations for formulation optimization were performed by reducing the ionizable lipid to mRNA weight ratio to 8 and 6, respectively for lipid A and lipid B and/or by using modified molar lipid compositions of DMG-PEG-2000: ionizable lipid A: cholesterol: DOPE (5:50:30:15) or DMG-PEG-2000: ionizable lipid B: cholesterol: DOPE (3:47:27.5:22.5). For all the formulations, the mRNA concentration in the feed stream of spray dryer was held constant at 0.33 mg/mL.

**Table 1:** Spray drying conditions for various solvents

Solvent	Inlet temperature (°C)	Aspirator (%)	Pump (%)	Outlet temperature (°C)
Water	90	85	25	46–50
20% Ethanol	60–65	85–100	15–25	28–35

### Characterization of mRNA-LNP DPP

The size of mRNA-LNPs was measured using Zetasizer (Malvern). Pre- and post-spray drying encapsulation of the mRNA was determined using standard ribogreen assay. The size of the DPP particles was measured using Mastersizer (Malvern). The mRNA was extracted from the DPP and its integrity was determined using capillary electrophoresis (CE, Agilent).

### Morphological analysis of mRNA-LNP DPP

Scanning electron microscopy (SEM) was used to visualize the DPP. Briefly, powder samples were sprinkled onto carbon adhesive tape that was mounted on SEM stubs. Excess powder was removed by blowing with clean compressed air. Prior to imaging, the powder samples were sputter coated with approximately 11 nm gold–palladium alloy in two cycles to avoid overheating.

#### *mRNA transfection *in vitro

HEK 293 cells were plated in 12 well plate format 24 h prior to transfecting at a concentration of 0.5 × 10^6^ cells/well. The DPP samples were reconstituted in OPTIMEM at 0.5 mg/mL mRNA concentration and then diluted to the required mRNA concentration before transfection. The protein expression was measured 24 h post transfection using ELISA. The cells expressing mCherry mRNA were counted using FACS.

### Animal studies

#### Intratracheal administration in mice

DPPs (approximately 2 mg of product) were administered intratracheally using Dry Powder Insufflator™ (Model DP-4 M PennCentury Inc., Wyndmoor, PA, USA) in 6 to 8 weeks old CD-1 male mice. Before intratracheal administration of dry powder, the mice were anaesthetized with isoflurane inhalation. While the animals were still anesthetized via a nose cone, the insufflator was intubated gently inside the trachea. The dry powder formulations were administered to the mice using a high-pressure syringe (Air Pump – Model AP-1; PennCentury Inc., Wyndmoor, PA, USA) and the powder was dispersed intratracheally with approximately 0.15 mL of air from the syringe. Before intratracheal liquid administration, the mice were anaesthetized with isoflurane inhalation to effect. While animals still anesthetized via a nose cone, a guiding catheter was intubated gently inside the trachea. The liquid formulations were administered to the mice intratracheally through the guiding catheter. For liquid aerosol administration, the 30 uL sample was administered via a pipette directly on the hub of the catheter while the animal slowly breathe it in and the animal is allowed to recover from the anesthesia and dosing. For the evaluations with FFLuc mRNA, 2 mg of DPPs (per animal) was dosed in animals as explained above. At 24 h post dosing, 3 mg of luciferin was administered in each animal via subcutaneous (SC) injection. Post euthanasia, different tissues were harvested and imaged in a petri dish using IVIS imaging (Perkin Elmer, USA).

#### Inflammatory and toxicity study

Mice were dosed with DPP and liquid formulations (saline and liquid LNP as control) as described above. For the LPS control group, 50 ug of LPS dissolved in 50 mL of PBS was administered per mouse. Following euthanasia, maximum obtainable volume of the whole blood was collected via cardiac puncture and analyzed for ALT/AST while the collected broncho-alveolar lavage fluid (BALF) was analyzed for cytokines and chemokines.

#### Single cell analysis

Single cell suspension for flow cytometry analysis was prepared as per the following method. Briefly, the mouse lungs were perfused with saline and then kept on wet ice. The lungs were then digested utilizing the GentleMACS octo-dissociator using the Lung Dissociation Kit (Miltenyi Biotec, Cat # 130–095-927). To stop the enzymatic digestion process, DMEM with 10% FBS was used, and the cell suspension was spun down at 500 g for 10 min. After which, the cell pellet was resuspended in 100 uL Cell Staining Buffer (Biolegend, Cat # 420,201) with Mouse FC Block (BD Pharmigen, Cat # 553,141) at a dilution of 1:10 for 10 min on wet ice. The cells were then stained for cell surface markers CD31 (Biolegend, Clone MEC 13.3), CD326 (Biolegend, Clone G8.8), CD45 (Biolegend, Clone 30-F11), and PDPN (Biolegend, Clone 8.1.1) for 30 min at 1:500 dilution. Cells were then fixed using Cyto-Fast™ Fix/Perm Buffer Set (Biolegend, Cat # 426,803) per manufacturer recommendation. Cells were then stained for intracellular marker TUBA (Novus, Clone DM1A). All analyses were carried out on a Cytek Aurora where viable (DAPI^Neg^ cells) were gated on singlets using FSC-A and FSC-H. Endothelial cells were defined as CD31^Pos^, CD45^Neg^, and CD326^Neg^. Leukocytes were defined as CD45^Pos^, CD31^Neg^, and CD326^Neg^. Epithelial cells were defined as CD326^Pos^, CD45^Neg^, and CD31^Neg^. Epithelial subtypes (i.e., CD326Pos, CD45^Neg^, CD31^Neg^) were categorized as follows: ciliated cells were TUBA^Pos^ and PDPN^Neg^, while type 1 cells were PDPN^Pos^ and TUBA^Neg^, and airway cells were epithelial cells defined as doubly negative for PDPN^Neg^ and TUBA^Neg^.

### Statistical analysis

A statistical test was carried out using Prism software version 6 (GraphPad Software Inc., San Diego, CA, USA) and evaluated using a Welch’s *t*-test. Differences were considered statistically significant at *p* < 0.05.

## Results and discussion

### Effect of changes in solvents on processing temperature and DPP characteristics

Although it was feasible to manufacture the DPP at the inlet temperature of 90 °C using water as a solvent, we reduced the process temperature to lower the mRNA degradation and to increase the yield by minimizing the adherence to the cyclone separator. As shown in Table 1, with 20% ethanol in the feed inlet stream, it was possible to reduce the inlet temperature to 60–65 °C and thus resulted in a lower outlet temperature of < 35C as desired. The presence of ethanol in the inlet feed also aided in reducing the particle size of the DPP. These results can be attributed to the lower operating temperature that was required to evaporate the hydroalcoholic solvent as compared to water. The lower temperature may have resulted in less melting of the lipids and prevented adhering as well as reduced aggregation, leading to an increase in yield and decrease in size.

### Effect of helper lipids and excipients on DPP characteristics

LNPs for the delivery of nucleic acids generally contain phosphatidylcholine (PC) or phosphatidylethanolamine (PE)-based helper lipids. We evaluated different headgroup helper lipids with higher phase transition temperatures to understand whether inclusion of these lipids could improve the yield by lowering the adherence to the cyclone separator. Hence, in addition to DOPE, other helper lipids such as DPPC and DEPE were evaluated with lipid A and lipid B LNP formulations at 1 mg/mL mRNA concentration, and 4% weight/volume mannitol. There were no significant differences in mRNA yield, LNP size, encapsulation, and DP size using DPPC and DEPE as compared to DOPE for lipid A. Lipid B formulations with DPPC or DEPE as helper lipids showed low mRNA encapsulation pre- and/or post-spray drying suggesting DPPC and DEPE were not compatible with ionizable lipid B in forming LNPs. Furthermore, for all of these formulations, the majority of the DP was adhered in the cyclone separator resulting in a very low yield in the collection vessel. These results suggest that other helper lipids like DPPC and DEPE did not provide any additional benefit in preventing adherence of the DPP to the cyclone separator and therefore, DOPE was selected as the helper lipid of choice for further evaluations.

Leucine as an excipient has shown improvement in powder characteristics along with enhancement in the yield by minimizing adherence to the cyclone separator. Several different amino acids such as leucine, trileucine, proleucine, glycine, and others were screened to determine the optimal excipient. None of the additional amino acids tested showed any benefits with respect to size, encapsulation, and yield over leucine; hence, leucine was selected as an excipient for further dry powder product development. The nomenclature of formulations refers to the amount of excipient per mg of mRNA. For example, a 20/10 mixture of mannitol:leucine refers to excipient mixture of 20 mg of mannitol and 10 mg of leucine per mg of mRNA in the DPP. All the formulations screened in this work are listed in Table 2 along with additional details such as the molar composition, lipid weight, excipients, and their amounts.

**Table 2:** List of formulations screened as part of DPP optimization and development. Columns represent the ionizable lipid used in the DPP formulation, the lipid molar composition, weight ratio of ionizable lipid/ mRNA, and the amount of mannitol and/or leucine per mg of mRNA in the formulation

Formulation	Ionizable lipid (A/B)	Molar composition (DMG-PEG 2 K: ionizable lipid A/B: cholesterol: DOPE)	Lipid A or B (mg) per mg of mRNA	Mannitol (mg) per mg of mRNA	Leucine (mg) per mg of mRNA
DPP-1	A	5:40:25:30	10	0	10
DPP-2	A	5:40:25:30	10	0	15
DPP-3	A	5:40:25:30	10	0	30
DPP-4	A	5:40:25:30	10	15	5
DPP-5	A	5:40:25:30	10	20	10
DPP-6	A	5:40:25:30	10	40	10
DPP-7	A	5:40:25:30	10	40	0
DPP-8	A	5:40:25:30	10	0	15
DPP-9	A	5:50:30:15	10	0	15
DPP-10	A	5:50:30:15	10	20	10
DPP-11	A	5:40:25:30	7	0	15
DPP-12	A	5:40:25:30	7	20	10
DPP-13	A	5:50:30:15	7	0	15
DPP-14	A	5:50:30:15	7	20	10
DPP-15	B	3:40:25:32	8	40	10
DPP-16	B	3:40:25:32	8	0	30
DPP-17	B	3:40:25:32	6	0	30
DPP-18	B	3:47:27.5:22.5	8	0	30

**Fig. 1:**
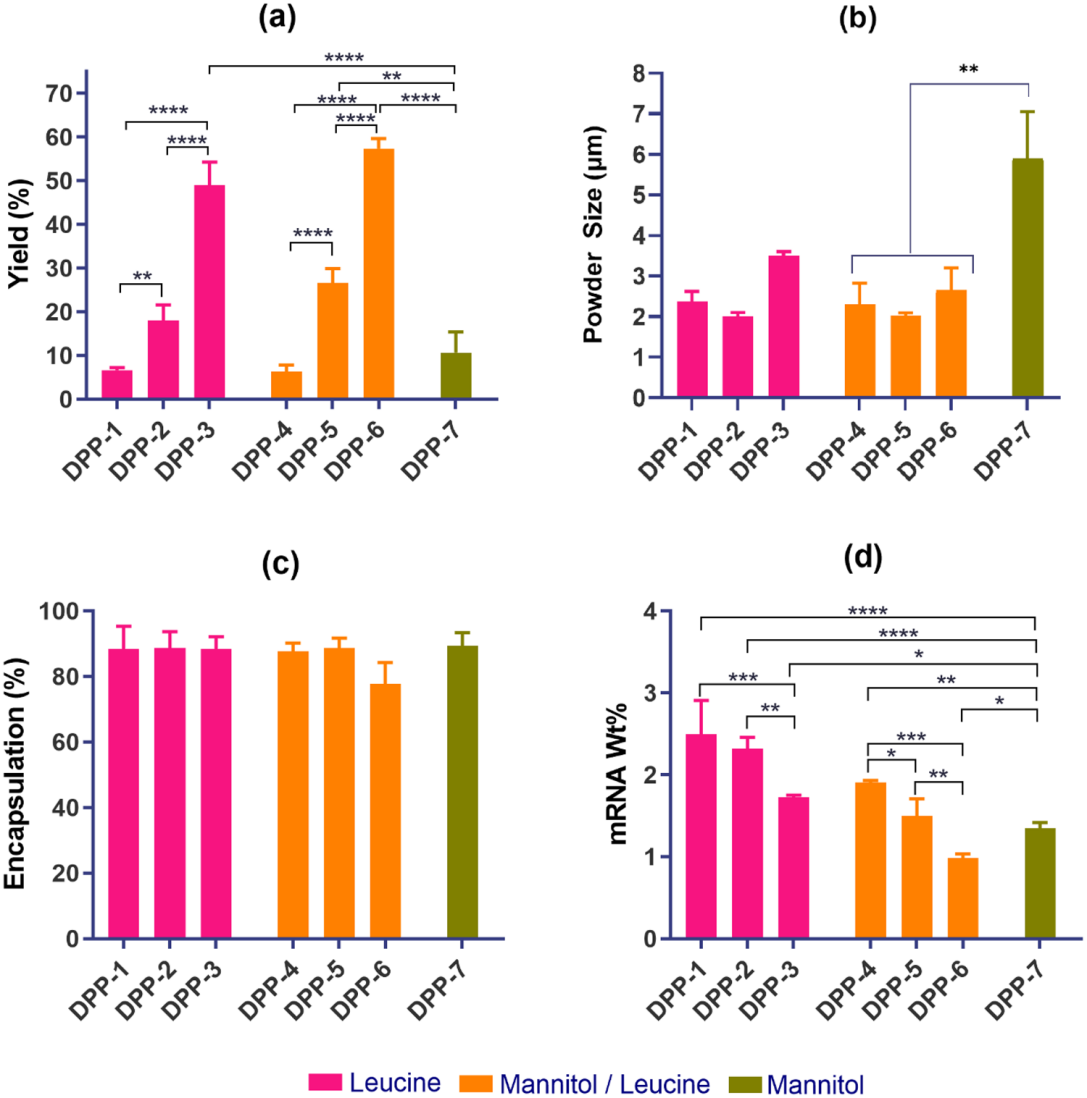
Effect of excipients on DPP characteristics (formulated with lipid A LNPs). **a** Yield in collection vessel, **b** DPP particle size, **c** mRNA encapsulation, and **d** mRNA weight percent in the DPP. Leucine alone or in combination with mannitol increased the yield and reduced the DPP particle size. mRNA encapsulation was not affected by excipients and mRNA weight percent decreased with increasing excipient amount. *p < 0.05, **p < 0.01, ***p < 0.001, ****p < 0.0001. Statistical significance was evaluated using a Welch’s *t*-test

For ease of discussion, the DPP formulation numbers from Table 2 are referred in the following sections. The effect of excipient amount on the DPP of lipid A at a constant amount of lipid content is shown in Fig. 1. Different DPPs (DPP-1 through DPP-7) with varying amounts of excipients were formulated using LNPs at molar ratio of DMG-PEG-2000: lipid A: cholesterol: DOPE (5:40:25:30). The size of the LNP formulations in this evaluation was between 60 and 80 nm. As shown in Fig. 1a, the use of leucine resulted in a significant improvement in yield. In the case of formulation with mannitol alone as an excipient (DPP-7), the majority of the DPP was lost in the cyclone separator with a very low yield of 10%. On the other hand, for formulation containing only leucine (DPP-3), the yield of DPP went up to 50% with good recovery in the collection vessel. For leucine alone as an excipient, increasing amount of leucine resulted in significant increase in yield. The highest yield of around 60% was obtained by employing a 40/10 mixture of mannitol/leucine (DPP-6). Furthermore, leucine alone and in combination with mannitol exhibited significant reduction in DPP particle size (Fig. 1b). DPP-7 formulated using only mannitol showed higher particle size of 6 µm, whereas DPP-1 through DPP-6, with leucine alone or in combination with mannitol, resulted in the desired size of < 4 µm. The observed increase in yield and decrease in DPP particle size can be attributed to the lower solubility in the hydroalcoholic solvent and the higher surface activity of leucine. The lower solubility of leucine could promote rapid coating of the newly forming DPPs and prevent adhesion resulting in higher yield. In addition, higher surface activity reduces cohesiveness which may have led to formation of DPP with smaller particle size. There were no significant differences in encapsulation for DPP formulated using mannitol, leucine, or the combination of leucine and mannitol (Fig. 1c). As expected, the measured mRNA weight percent decreased with the increase in excipient amount (Fig. 1d). Given the advantages of mannitol as described above and the improvements with addition of leucine, we next focused our evaluations on leucine and mannitol combinations as excipients. Although increasing the excipient amount increased the yield, it decreased the mRNA weight percent in the DPP. Hence, to optimize the balance of higher yield and sufficient mRNA content in the DPP, lipid content of the LNP was varied in subsequent evaluations.

### Effect of lipid amount on DPP characteristics

**Fig. 2:**
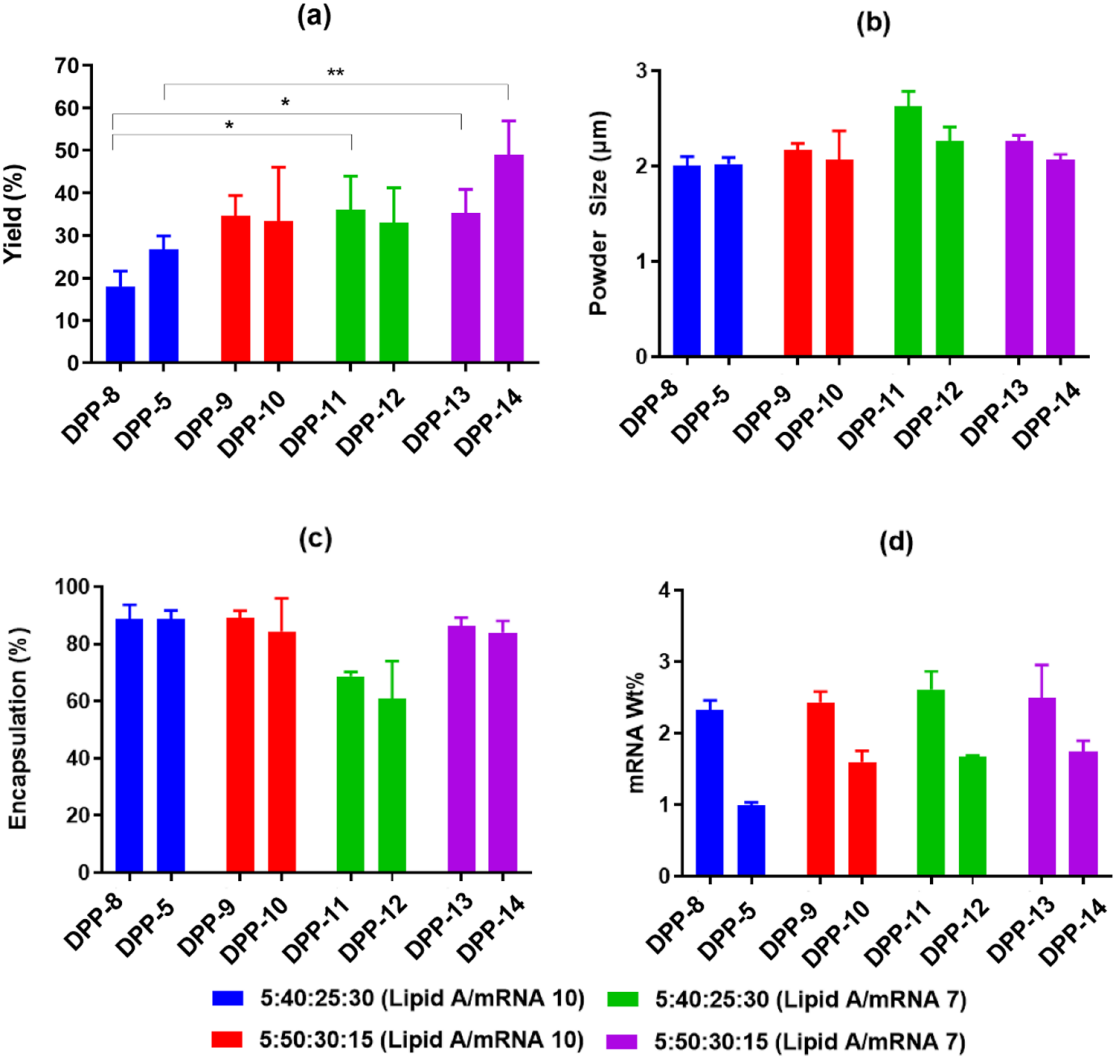
Effect of lipid amount on DPP characteristics. **a** Yield in collection vessel, **b** DPP particle size, **c** mRNA encapsulation, and **d** mRNA weight percent in the DPP. Total lipid content was reduced by either by changing the LNP composition or by decreasing the lipid A to mRNA weight ratio. Overall, the yield and mRNA weight percent were increased with lowering the lipid amount with minimal changes in DPP powder size and LNP encapsulation. *p < 0.05, **p < 0.01. Statistical significance was evaluated using a Welch’s *t*-test

The reduction in the total lipid content of the LNPs was achieved by either lowering the weight ratio of ionizable lipid to mRNA and/or changing the composition by increasing the mole percent of the ionizable lipid. Figure 2 represents the effect of lowering lipid content on the DPP manufactured using leucine alone or in combination with mannitol. The DPP yield was improved with lowering the total lipid amounts for leucine alone as well as mannitol/leucine combination products (Fig. 2a). As an example, for the formulation DPP-13 containing 15 mg leucine/mg of mRNA, with lipid A/mRNA weight ratio of 7 and a modified lipid composition of DMG-PEG-2000: lipid A: cholesterol: DOPE (5:50:30:15), the yield was more than 30% as compared to less than 20% yield for DPP-8 with LNPs at lipid A/mRNA weight ratio of 10 and lipid composition of DMG-PEG-2000: lipid A: cholesterol: DOPE (5:40:25:30). Similarly, higher yield of around 50% was obtained for DPP-14 formulation with 20/10 mg of mannitol/leucine per mg of mRNA formulated using LNPs at a lipid A/mRNA weight ratio of 7 and a modified lipid composition of DMG-PEG-2000: lipid A: cholesterol: DOPE (5:50:30:15) as compared to a yield of about 30% for LNPs at a lipid A/mRNA weight ratio of 10 and lipid composition of DMG-PEG-2000: lipid A: cholesterol: DOPE (5:40:25:30). The reduction in lipid amount may have resulted in a lower degree of melting and consequently, less adhesion tendency that led to increase in the yield. For most formulations with lower lipid content, there was no significant difference in encapsulation efficiency (Fig. 2c), and hence, we were able to achieve the optimal balance of improving yield by lowering lipid amounts without affecting the mRNA encapsulation. The particle size was in the desired range of less than 3 µm for all the DPPs (Fig. 2b). Hence, we were able to improve the yield without affecting other critical parameters like encapsulation and weight % by optimization of lipid amounts in the LNPs.

### Effect of excipients and lipid amount on DPP morphology

**Fig. 3:**
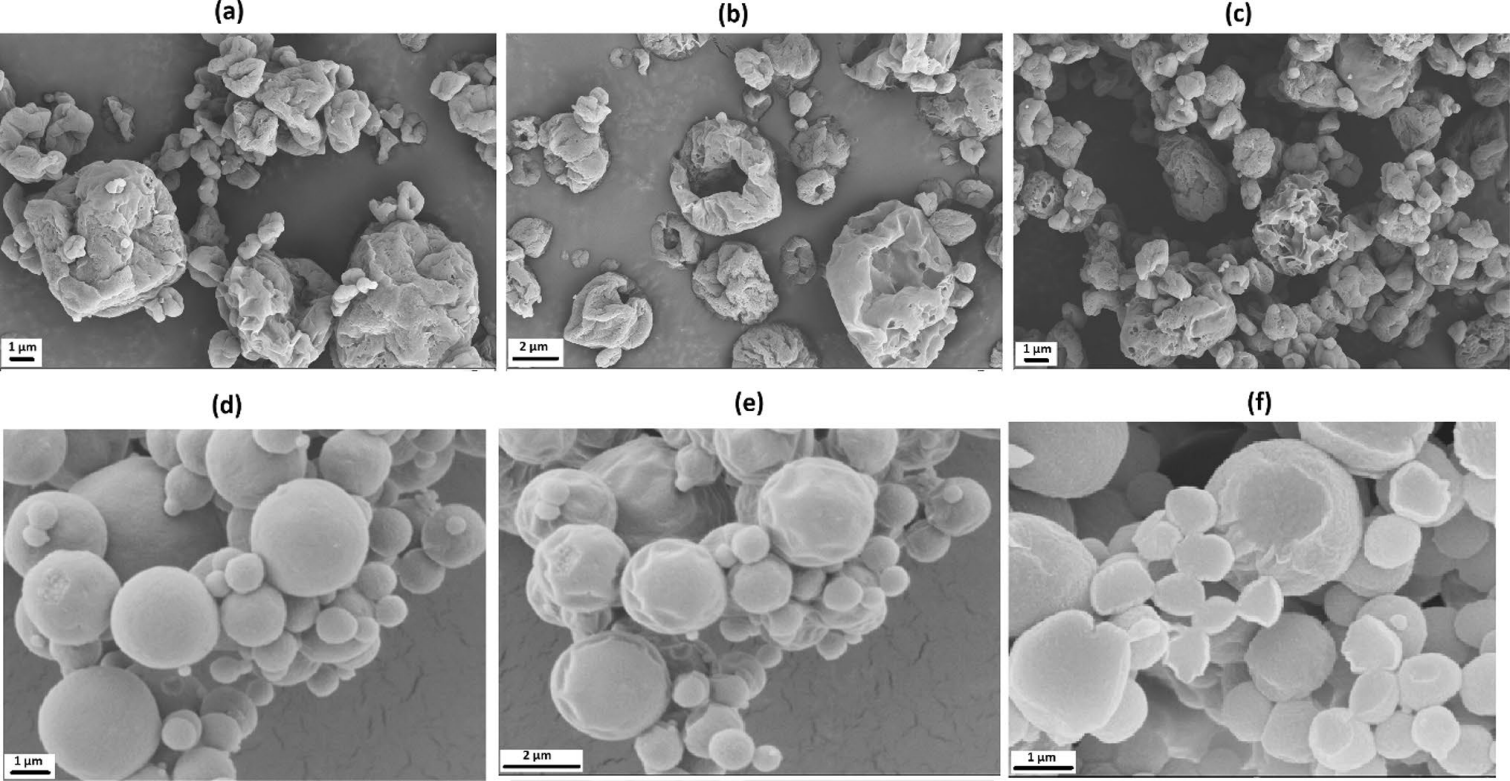
SEM images for some of the representative DPPs formulated using lipid A LNPs. **a** DPP-2: leucine 15 mg only at lipid A/mRNA weight ratio 10, **b** DPP-11: leucine 15 mg only at lipid A/mRNA weight ratio 7, **c** DPP-13: leucine 15 mg only with modified LNP composition at lipid A/mRNA weight ratio 7, **d** DPP-6: mannitol 40 mg and leucine 10 mg combination with regular LNP composition at lipid A/mRNA weight ratio 10, **e** DPP-6: exposed for longer duration, and **f** DPP-6: cross-section image. DPPs of leucine alone showed wrinkled and collapsed appearance with a significant porosity, whereas DPPs with mannitol and leucine combination appeared smoother and spherical with a solid core

Scanning electron microscopy (SEM) images for select representative DPPs formulated using lipid A are shown in Fig. 3. Similar surface characteristics were observed for lipid B DPP (data not shown). DPP-2 formulated using leucine alone showed particles with wrinkled and collapsed appearance with a significant porosity (Fig. 3a). As shown in Fig. 3b and c, DPP-11 and DPP-13 with different lipid A: mRNA weight ratio as well as modified LNP composition showed similar appearance indicating no significant change in the surface characteristics because of the lowering of the lipid content of the LNPs. DPP-6 made using a leucine-mannitol combination appeared smoother and more spherical (Fig. 3d). The surface of these particles showed slight indentations, wrinkles, and dimples because of damage due to longer exposure to the electron beam as shown in Fig. 3e. Cross-section images of these particles appeared to have a solid core (Fig. 3f). The spherical morphology of the mannitol and leucine combination DPP can be attributed to the higher excipient content as well as to the presence of mannitol that may have provided higher amount of material to form a matrix.

### Effect of spray drying process onmRNA integrity and in vitro protein expression

**Fig. 4:**
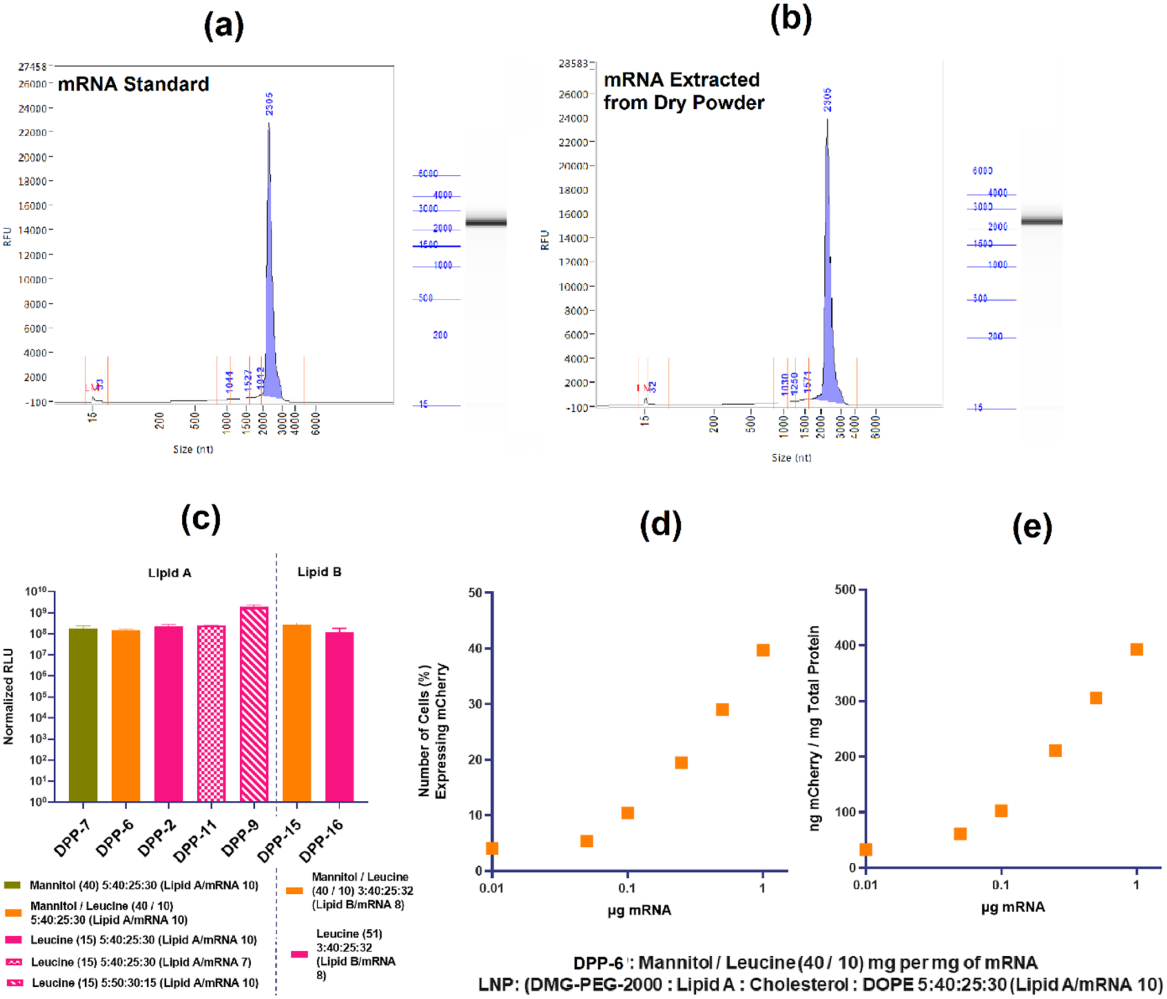
Capillary electrophoresis (CE) showing mRNA integrity of **a** FFL mRNA standard and **b** mRNA extracted from DPP. No significant differences indicate mRNA integrity is retained post spray drying. **c** In vitro FFL expression in HEK-293 cells for various DPPs. Dose-dependent protein expression of mCherry DPPs showing **d** number of cells expressing mCherry protein and **e** amount of mCherry protein expressed by the cells. No significant loss in mRNA integrity due to spray drying process and successful dose-dependent in vitro transfection suggests feasibility of the DPP as mRNA delivery system

Considering the transient exposure of LNPs to heat and stress during spray drying, one of the most critical evaluations of the developed DPP is mRNA integrity post spray drying. As shown in Fig. 4, mRNA extracted from the DPP (Fig. 4b) resulted in a superimposable peak as compared to the control mRNA standard (Fig. 4a) with no significant difference in the mRNA integrity as measured using capillary electrophoresis (CE). All of the DPPs formulated using FFLuc mRNA demonstrated successful in vitro transfection. HEK293 cells treated with different DPPs (DPP-2, DPP-6, DPP-7, DPP-9, DPP-11, DPP-15, and DPP-16) formulated with various combinations of excipients and varying lipid content resulted in successful protein expression of Renilla luciferase (Renilla Luc) mRNA as measured by luminescence (Fig. 4c). To rule out an mRNA protein expression bias, mCherry encoding mRNA DPP was employed. mCherry is a fluorescent protein that is utilized extensively in research, it has superior signal to background ratio and minimal photobleaching properties [35], making it ideal for protein expression assessment. To this end, HEK293 cells treated with mCherry DPP resulted in a dose-dependent protein expression. The number of mCherry positive cells (Fig. 4d) and the amount of mCherry protein expressed over total cellular protein (Fig. 4e) increased with increasing amount of DPP mRNA. These initial experiments demonstrated no significant loss in mRNA integrity due to the spray drying process. Successful in vitro protein expression provided support for the feasibility of the DPP applications in vivo. Based on these promising results, lead DPPs formulated with various combinations of excipients and lipids were selected for in vivo evaluations.

### Effect of ionizable lipid, mRNA loading, and excipients on in vivo expression

**Fig. 5:**
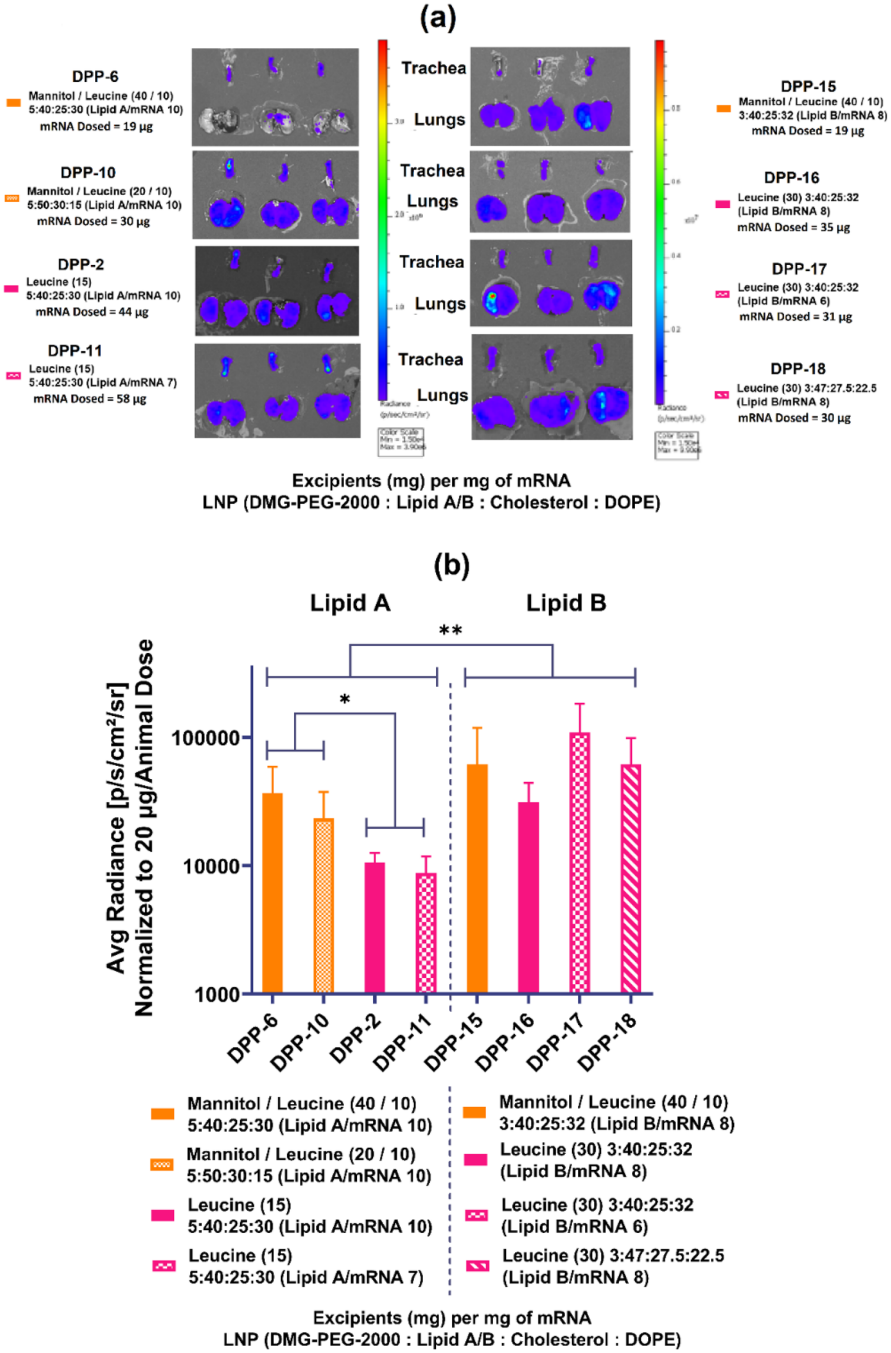
Pulmonary delivery of mRNA with different DPP formulations. Screening of DPPs in CD-1 mice via intratracheal administration. **a** IVIS images of trachea and lungs showing uniform luminescence at corresponding mRNA doses based on their individual mRNA weight percent and **b** plot of average radiance of luminescence measured in trachea and lungs normalized to 20 µg of mRNA dose. FFL expression for lipid A DPPs was significantly increased by using mannitol and leucine in combination rather than using leucine alone or by reducing the lipid content. *p < 0.05, **p < 0.01. Statistical significance was evaluated using a Welch’s *t*-test

As shown in Fig. 5a, all FFLuc mRNA containing DPPs using lipid A or lipid B showed bioluminescence in the trachea and lungs of the mice suggesting successful delivery with sufficient deposition, sedimentation, and subsequent absorption of the functional DPP with desired particle size (< 4 µm) in the lungs. The lower solubility of leucine in hydroalcoholic solvent and its higher surface activity may have led to formation of low density corrugated DPP particles with an improved aerosol performance. The comparison of average radiance normalized to 20 µg mRNA dose per animal for the DPPs of LNPs made using lipid A or lipid B is shown in Fig. 5b. For lipid A DPPs, combination of excipients resulted in significant changes in protein expression. Overall, protein expression for lipid A DPPs was significantly increased by using mannitol and leucine in combination (DPP-6 and DPP-10) rather than using leucine alone (DPP-2 and DPP-11). As an example, no significant difference in average radiance was detected for DPPs (DPP-2 and DPP-11) at two different lipid A to mRNA weight ratios (ratio 10 and ratio 7) manufactured using leucine alone. Changes in LNP composition did not show any significant difference in average radiance for mannitol-leucine combination. On the other hand, cumulative average radiance of both mannitol-leucine DPPs was higher as compared to that of leucine-based DPPs. This higher expression with mannitol-leucine combination for lipid A DPP may be attributed to its spherical morphology (Fig. 3d) and consequently better aerosolization, dispersibility, and deposition in the lung tissue. No statistically significant difference in average radiance was detected by changing excipients or lipid amounts for lipid B DPPs. Based on all the above findings, we see DPP-10 formulation of lipid A with mannitol/leucine (20/10) and molar composition of 5:50:30:15 (DMG-PEG 200, lipid A, cholesterol, DOPE) shows the optimal balance in terms of evenly distributed in vivo protein expression (Fig. 5a), encapsulation rate (Fig. 2c), weight percent (Fig. 2d), and yield (Fig. 2a), leading to its selection for additional evaluations.

**Fig. 6:**
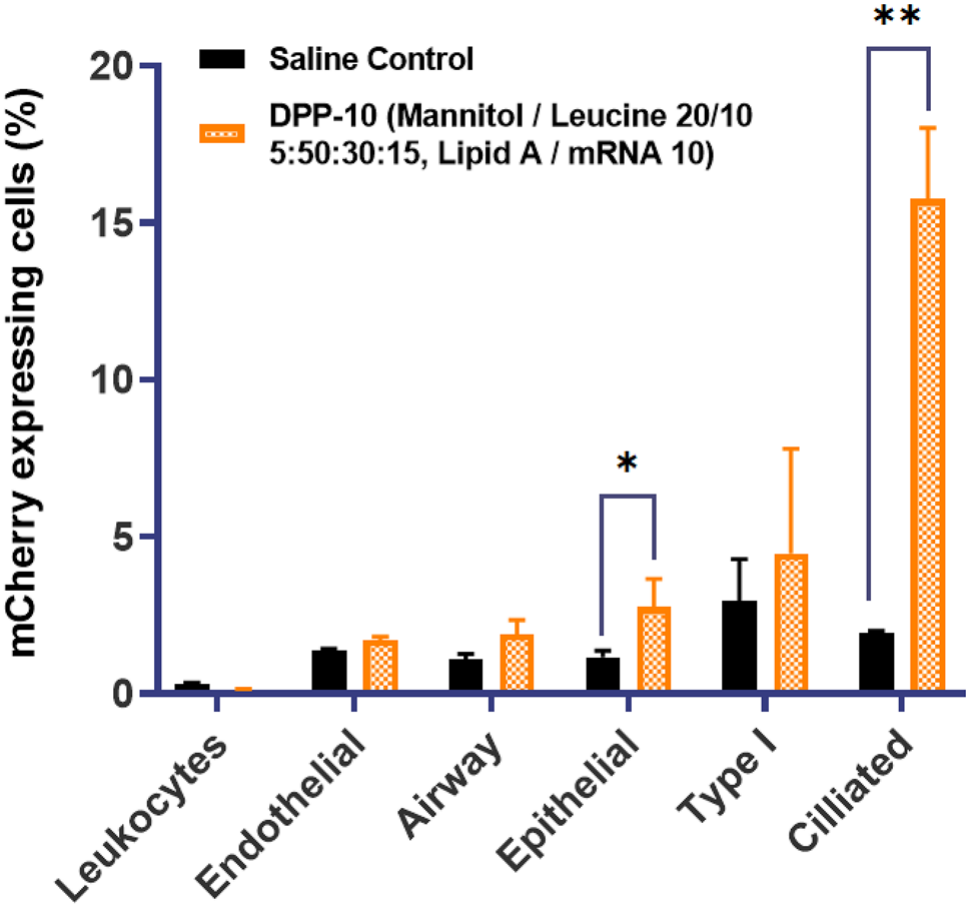
Flow cytometry analysis of murine lungs demonstrate mCherry expression predominantly in epithelial cells. Expression is enriched in ciliated epithelial cells when compared to airway and Type 1 cells. *p < 0.05, **p < 0.01. Statistical significance was evaluated using a Welch’s *t*-test

As shown in supplementary Fig. S2, for the lead DPP-10 formulation, biodistribution study was performed to evaluate if there is any uptake in off target organs. As expected and desired, for both the liquid and the DPP formulation, protein expression was observed only in the trachea and lungs. No significant expression in the liver, spleen, kidney, heart, and reproductive organs was detected, thus confirming the intended delivery. Like the biodistribution results for the DPP, the corresponding liquid control formulation also showed signal only in the trachea and lungs (data not shown). Single cell analysis of DPP-10 was also conducted to understand the specific cell types that are transfected. As can be seen from Fig. 6, flow cytometry analysis demonstrated minimal uptake by leukocyte and endothelial cells. Low expression in leukocytes could indicate favorable profile in terms of the formulation being less immunogenic. Epithelial cells were the predominant cell type that expressed mCherry. Like previous findings in the context of pulmonary delivery, ciliated epithelial cells were the predominant epithelial subtype that expressed mCherry [36]. Successful delivery of mRNA and protein expression in multi ciliated cells is of great significance for treatment of diseases such as primary ciliary dyskinesia (PCD), thereby indicating therapeutic potential for this formulation.

### Safety profile of DPP

**Fig. 7:**
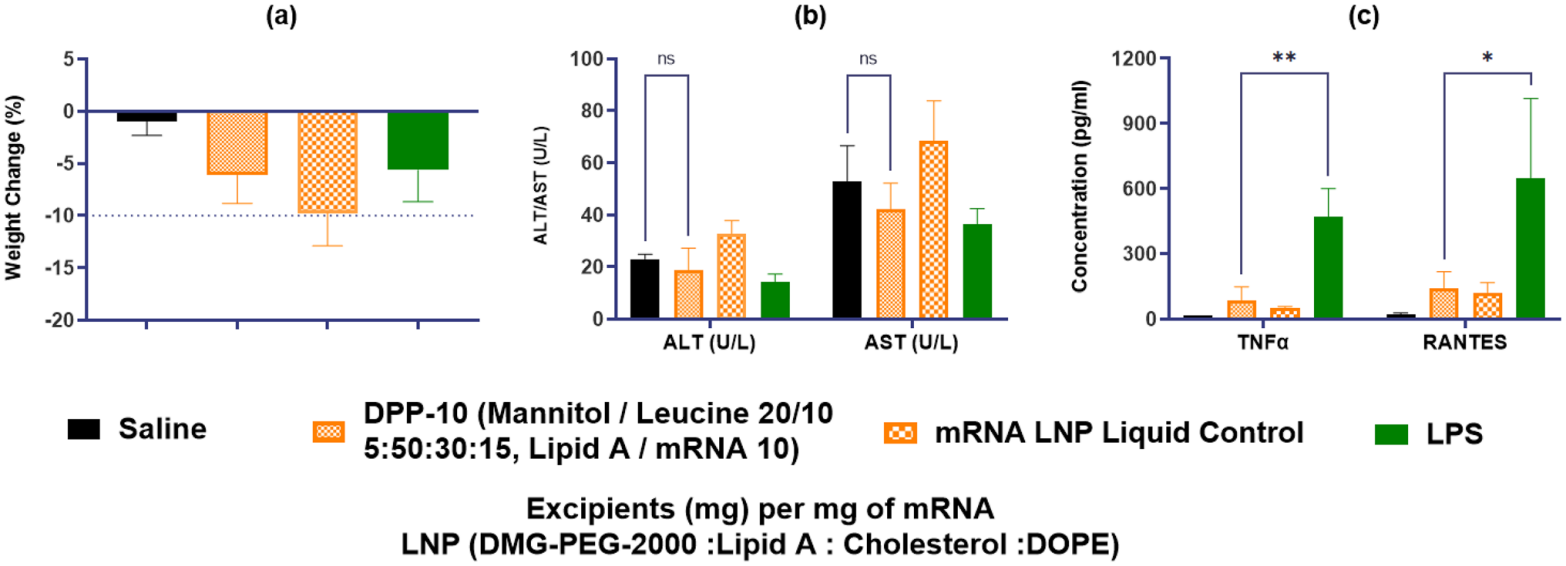
Safety evaluation of the formulations in mice. CD-1 mice were administered intratracheally with saline as control; LPS (50 µg in 50 µl per animal); DPP-10 (2 mg containing 30 µg of mRNA equivalent per animal); mRNA LNP liquid control (30 µg of mRNA equivalent in 50 µl per animal). **a** Body weight of the mice was monitored before and at 24 h post administration of the formulation. **b** ALT and AST levels were measured at 24 h post administration. **c** Levels of pro-inflammatory cytokine and chemokine: TNF-alpha and RANTES were measured at 24 h post administration of the formulations. *p < 0.05, **p < 0.01. Statistical significance was evaluated using a Welch’s *t*-test

Finally, for the optimized “lead” DPP-10 formulation, some basic evaluations to assess the safety profile of the DPP were performed. As can be seen from Fig. 7a, dosing of the DPP formulation or the corresponding mRNA LNP liquid formulation (control) did not cause any significant decrease in body weight at 24 h post administration. Body weight decrease was < 10% for the DPP-10 formulation. Similarly, there was no significant elevation in ALT/AST enzymes for the formulation groups (Fig. 7b). This aligns with the biodistribution study results wherein we do not observe distribution to off target tissues including liver. The inflammatory response of the formulations was evaluated by measuring the level of pro-inflammatory cytokines and chemokines in BALF. As can be seen from Fig. 7c, elevations in TNF-alpha and RANTES were observed for the LPS group used as control, while the formulation groups (both the mRNA LNP liquid control and the DPP-10) remained non-inflammatory. In addition, other interleukins like IL-2, IL-4, IL-7, IL-10, and IL17 were not affected by the formulations (data not shown). Also, no significant difference between the DPP-10 and the liquid control was detected indicating that the dry powder did not affect the safety profile. Overall, the optimized DPP-10 formulation did not show any significant signs of inflammation or toxicity indicating a favorable safety profile.

From the above findings, we demonstrated that formulation optimization resulted in identification and development of a lead mRNA LNP-DPP formulation which, in turn, significantly improved the dry powder characteristics and efficacy of delivery while showing a favorable safety profile.

## Conclusion

In summary, we have established the successful development of dry powder formulations of mRNA LNPs suitable for inhalation. Optimization of formulations was achieved through evaluation of different excipients, solvents, lipid/mRNA concentration, processing parameters, and composition of LNPs. Combination of leucine and mannitol as excipients resulted in an optimal formulation which provided favorable characteristics. Intratracheal administration of these novel optimized formulations resulted in effective deposition and transfection in the lungs confirming successful delivery of the intact functional mRNA. In vivo evaluations of the lead formulation demonstrated expression in the desirable epithelial and ciliated cells and a favorable safety profile with low risk of inflammatory response and toxicity. This work demonstrates the potential and promise of developing a dry powder formulation of mRNA LNP with broad therapeutic applications for lung delivery.

### Supplementary Information

Below is the link to the electronic supplementary material.

Supplementary file1 (DOCX 427 KB)

## Data Availability

The raw data required to reproduce these findings may be available upon request. The processed data required to reproduce these findings may be available upon request.
