# Supplementary material for: Inhalable dry powder product (DPP) of mRNA lipid nanoparticles (LNPs) for pulmonary delivery
Source: Drug Deliv Transl Res. 2023 Aug 1;14(2):360–72. doi: 10.1007/s13346-023-01402-y (PMC10761450; doi:10.1007/s13346-023-01402-y)
Supplement: Supplementary file 1 — Supplementary file1 (DOCX 427 KB) [file 13346_2023_1402_MOESM1_ESM.docx]

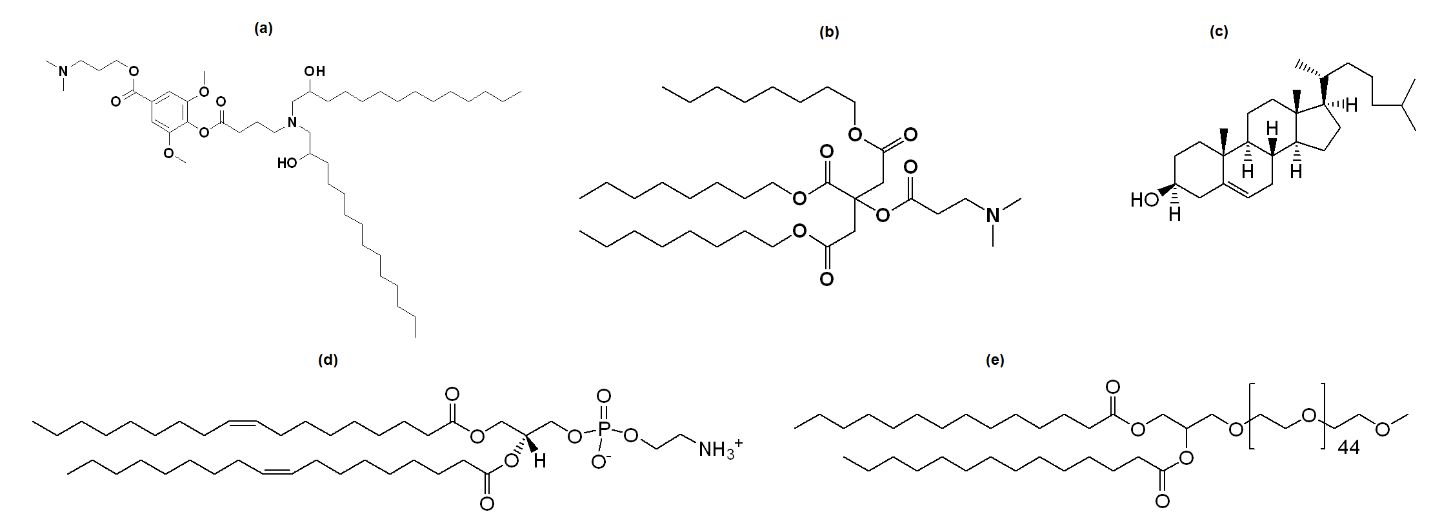


### Figure S1. Lipid components of LNP formulations (a) Novel ionizable lipid A based of phenolic acid core (b) Novel ionizable lipid B based of citric acid core (c) Cholesterol (d) 1,2-dioleoyl-sn-glycero-3-phosphoethanolamine (DOPE) (e) 1,2-dimyristoyl-rac-glycero-3-methoxypolyethylene glycol-2000 (DMG-PEG 2000)

###
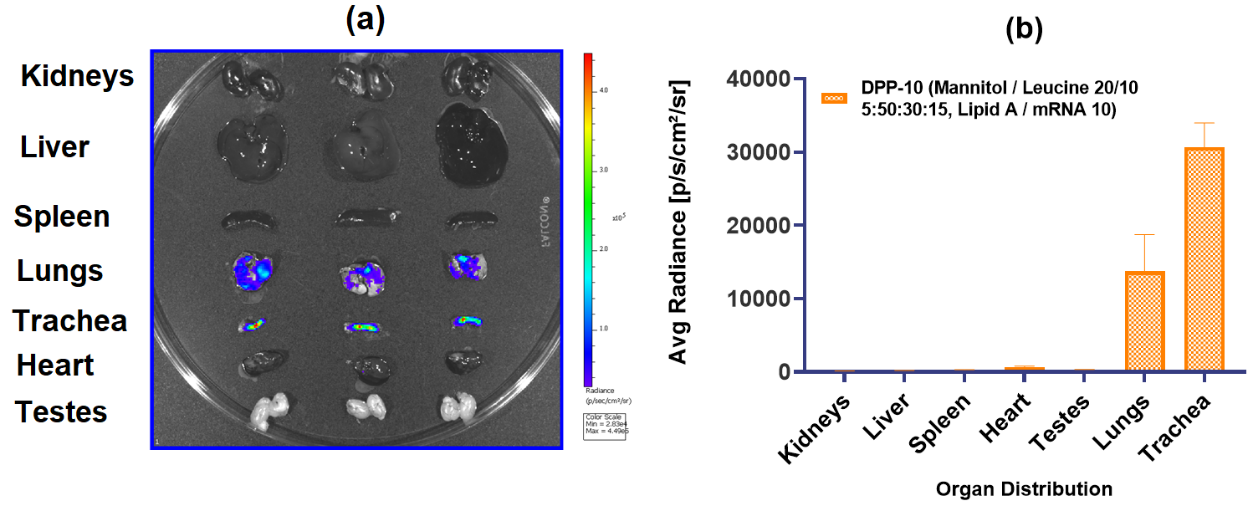


### Figure S2. Biodistribution evaluation of DPP-10. CD-1 mice were administered intratracheally with DPP (2 mg containing 30 µg of mRNA equivalent per animal). Post euthanasia different tissues like kidney, liver, spleen, heart, testes, lungs, and trachea were harvested 24 hours after the intratracheal dosing to image for luminescence using IVIS (a) IVIS images show signal primarily in trachea and lung tissues (b) Luminescence average radiance for different tissues. All images are on the same scale. Max: 4.5e5 and Min: 2.8e4 (p/sec/cm2/sr).
